# Hand Size Measurements in Children Aged 1–15 Years to Help the Development of Pediatric Electromyography Sensors for Neuromuscular Monitoring

**DOI:** 10.3390/jcm14217462

**Published:** 2025-10-22

**Authors:** Réka Nemes, Erzsébet Németh, Katalin A. Szatmári, Adrienn Timkó, Péter Luterán, Sorin J. Brull, Béla Fülesdi, Adrienn Pongrácz

**Affiliations:** 1 Department of Anaesthesiology and Intensive Care, University of Debrecen, 4032 Debrecen, Hungary; 2 Edömér Tassonyi Neuromuscular Research Group, 4032 Debrecen, Hungary; 3 Department of Anesthesiology and Perioperative Medicine, Mayo Clinic College of Medicine and Science, Jacksonville, FL 32224, USA

**Keywords:** electromyography, neuromuscular block, neuromuscular monitor, pediatric neuromuscular monitoring

## Abstract

**Background/Objectives:** The aim of this observational study was to collect hand measurements and anthropometric data in children aged 1–15 years of age to help the design of a pediatric skin electrode for electromyography-based neuromuscular monitoring. **Methods:** Data collection was performed at the Pediatric Department of the University of Debrecen Medical Centre between 1 December 2019 and 31 January 2021. After gaining written informed consent from the parents or legal representatives and verbal acceptance from age-appropriate (12–35 months) patients, a total of 153 children were enrolled. The following parameters were recorded: demographics (age, sex, weight, height, and hand dominance) and hand size parameters, defined as the distance between the following reference points: the ulnar groove and the midpoint of the hypothenar eminence (A); the midpoint of the hypothenar eminence and the first interphalangeal joint of the 5th finger (B); the ulnar groove and the midpoint of the thenar eminence (C); the midpoint of the thenar eminence and the interphalangeal joint of the thumb (D); the midpoint of the wrist crease and the tip of the third finger; wrist circumference (E); and forearm length. All measurements were made in centimeters (cm). **Results:** The children were divided into 4 groups (12–23 months, 2–5 years, 6–11 years and 12+ years). The number of children in the groups ranged between 6 and 16. The hand size parameters increased according to the children’s age (A: 4.3 ± 0.4, 5.0 ± 0.7, 6.3 ± 0.6, and 6.9 ± 1.0 cm; B: 3.2 ± 0.4, 4.1 ± 0.7, 5.0 ± 0.6, and 5.9 ± 0.6 cm; C: 3.0 ± 0.3, 3.6 ± 0.7, 4.1 ± 0.6, and 4.9 ± 0.6 cm; D: 4.1 ± 0.4, 4.8 ± 0.8, 6.2 ± 0.8, and 7.2 ± 0.9 cm; E: 10.1 ± 0.6, 12.0 ± 1.1, 15.3 ± 1.3, and 17.7 ± 1.7 cm, respectively, in the four groups, [mean ± SD]). The height of the children showed a closer correlation with hand size parameters (Pearson’s correlation coefficients: 0.702–0.961) than with age (0.665–0.904) or weight (0.675–0.863). The correlation was weaker when data were examined in prespecified age groups. **Conclusions:** The current pediatric hand size database provides previously unavailable information that was used in one manufacturer’s design, which may help with the future design of pediatric electrodes of electromyography-based neuromuscular monitors; this information may facilitate adoption of quantitative neuromuscular monitoring in routine pediatric anesthesia practice.

## 1. Introduction

In recent years, the topic of intraoperative neuromuscular monitoring has become increasingly important, resulting in the publication of consensus statements [1,2] and clinical guidelines [3,4]. It has been recognized that postoperative residual neuromuscular block is an independent risk factor for postoperative complications like desaturation, pulmonary aspiration and pneumonia. Additionally, appropriate intraoperative management of neuromuscular block using quantitative monitoring improves surgical conditions and patient outcomes [3].

Appropriate perioperative neuromuscular block management cannot be achieved without reliable objective (quantitative) neuromuscular monitors. Until recently, such monitors were not available or used widely in the clinical setting in adult patients. Quantitative monitors were even more difficult to access for clinical use in pediatric patients, rendering intraoperative neuromuscular monitoring of the pediatric patient challenging—and frequently neglected.

To answer these clinical needs, medical device manufacturers have started to develop new user-friendly quantitative monitors that use different measuring modalities. Some monitors use acceleromyography (AMG), while others use electromyography (EMG). Regardless of the measuring technology, the concept of quantitative neuromuscular monitoring is the same: a peripheral nerve that is easily accessible is stimulated via skin surface electrodes, and the resultant muscle response is recorded from the target innervated muscle. When AMG is used, the piezoelectric sensor (accelerometer) is attached to the adductor pollicis (AP) muscle, which measures the acceleration of the thumb contraction in response to ulnar nerve depolarization provided by a set of stimulating (positive and negative) skin electrodes [5]. When EMG is used, nerve stimulation is achieved at the same site (ulnar nerve on the distal volar forearm), and the elicited compound muscle action potential (cMAP) can be recorded via an active electrode placed on the belly of the target muscle, and a reference electrode placed on the muscle’s insertion point [5]. The most common recording of EMG can be achieved at the AP, abductor digiti minimi (ADM), or first dorsal interosseous (FDI) hand muscles. Although AMG has long been the technology used most commonly in the clinical setting, it has significant limitations. First, the AMG response in the unparalyzed patient results in significant overshoot in the TOF ratio (110% up to 140%), a ratio that is physiologically improbable [6]. Second, the variability of AMG responses at baseline is far greater than the variability of other measurement technologies such as EMG [6]. Third, AMG measurement of muscle function is based on movement, thus restricting its application in surgical cases in which the arms are placed under surgical drapes (e.g., laparoscopic, robotic, prone position), which limit thumb movement. To design appropriate EMG sensors for children of different ages, it is crucial to determine the correct dimensions of the forearm and hands, but pediatric anthropometric data to help design appropriately sized electrodes are scarce.

Current neuromuscular monitoring guidelines recommend assessing neuromuscular function of the AP muscle evoked by ulnar nerve stimulation. In the clinical setting, however, many clinicians prefer to record responses from the ADM or from the FDI muscles. Therefore, to facilitate the development of pediatric hand strip electrodes of EMG-based neuromuscular monitors (such as the TetraGraph, Senzime AB, Uppsala, Sweden) we collected hand size measurements and anthropometric data in children aged 1–15 years.

## 2. Methods

The presentation of data is in accordance with the STROBE (STrengthening the Reporting of OBservational studies in Epidemiology, www.strobe-statement.org) guidelines.

### 2.1. Ethics

Ethical approval for this study (DE RKEB/IKEB 5423-2019) was provided by the Research Ethics Committee of the University of Debrecen, Debrecen, Hungary (Chairperson: József Szentmiklósi) on 27 May 2019.

### 2.2. Patient Enrollment

Data collection was performed at the Pediatric Department of the University of Debrecen Medical Centre between 1 December 2019, and 31 January 2021. Patients and their parents were interviewed and were counseled/informed about the study aims and procedure during their hospital intake interview in the ward. The measurements required 5–10 min. Data were recorded on a data entry form that included the children’s height, weight, age and handedness, but was otherwise de-identified in order to protect the anonymity of the patients’ Personal Health Information (PHI). After collection, all data forms were stored in a secure location for 25 years, after which the forms will be destroyed. Patients with upper extremity injuries or deformities that would preclude hand measurements were excluded, as were patients who declined participation in the study.

**Figure 1:**
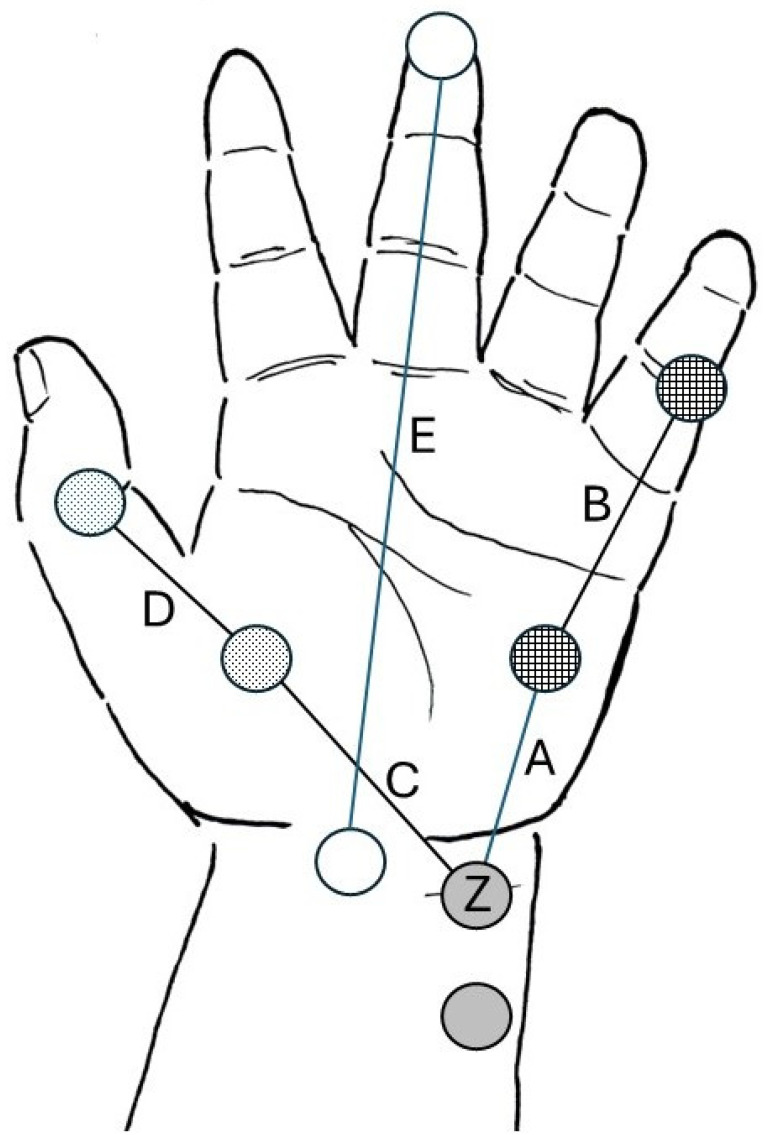
Depiction of the various distances between pre-determined anatomic reference points of the hand. Gray circles represent the ideal electrode positioning of stimulating electrodes. Z represents the location of the distal (negative) stimulating electrode. Checkered circles represent the ideal position of the active sensing electrode over the belly of the abductor digiti minimi muscle at the hypothenar eminence, and the reference electrode is placed over the first interphalangeal joint of the fifth finger. Dotted circles represent the ideal positioning of the active sensing electrode over the belly of the adductor pollicis muscle at the thenar eminence and the reference electrode is placed over the interphalangeal joint of the thumb. A, B, C, D segments are the specific inter-electrode distances that need to be considered when designing the pediatric strip electrode. Empty circles (E segment) represent the distance between the midpoint of the wrist crease and the tip of the third finger (hand length). Illustration courtesy of Senzime AB (publ.), Uppsala, Sweden.

After obtaining written informed consent from the parents or legal representatives as well as verbal acceptance from age-appropriate patients, a total of 153 children were enrolled. Afterwards, the following parameters were recorded in the clinical research form that did not contain any identifiable data: demographics (age, sex, weight, height, hand dominant side); hand size parameters, defined as the distance between: the ulnar groove just proximal to the wrist crease (Z) and the midpoint of hypothenar eminence (A); the midpoint between mid-hypothenar eminence and first interphalangeal joint of the 5th finger (B); the ulnar groove just proximal to the wrist crease (Z) and midpoint of the thenar eminence (C); the midpoint of the thenar eminence and the interphalangeal joint of the thumb (D); the midpoint of the wrist crease and tip of the third finger (E) (Figure 1); wrist circumference; and forearm length. Specific distances were measured in both hands, using a 1.5-m soft tape measure (Prym Group, Stolberg, Germany). All measurements were performed in duplicate to ensure accuracy, and the average values were recorded.

Distances A and C represent the distance between the location of the distal negative stimulating electrode (Z) at the wrist and the active sensing electrodes at the abductor digiti minimi and adductor pollicis muscles, respectively. Distances B and D represent the distance between the active sensing electrodes of the abductor digiti minimi and adductor pollicis muscle, respectively, and the corresponding distal reference electrodes.

### 2.3. Statistics

This was an exploratory descriptive study, and no previous similar pediatric hand measurements could be identified in the literature. Therefore, no power analysis was performed a priori. Basic descriptive statistics (Pearson’s correlation) were used to describe the data; *p* < 0.05 was used to define statistical significance. SigmaPlot for Windows software package (version 11.0, Chicago, IL, USA) was used for statistical calculations.

## 3. Results

A total of 153 children were enrolled in the study. Fully complete datasets were obtained from 126 children. The number of children in each year group varied between 6 and 16.

The hand size parameters increased according to the children’s age as shown in Table 1. Height of the children showed closer correlation with hand size parameters (Pearson’s correlation coefficients: 0.702–0.961) than age (0.665–0.904) or weight (0.675–0.863) (Table 2). Correlation was weaker when the data were examined in prespecified age groups (12–23 months, 2–5 years, 6–11 years, and 12–15 years, Table 2). The analysis of the differences between the 4 grouped age bins (1–2 years old; 2–5 years old; 6–11 years old; and 12–15 years old) used ANOVA with Tukey, and presents the F-statistic, *p*-value and eta-squared results in Table 3. The data were analyzed with the help of ChatGPT-5o (OpenAI). As expected, all variables showed highly significant differences across the four age bins (*p* < 0.001). Eta-squared values are large for most variables, indicating a large proportion of variance can be explained by the age-bin factor (as expected for growing children).

## 4. Discussion

Appropriate perioperative neuromuscular block management cannot be achieved without reliable objective (quantitative) neuromuscular monitors. Until recently, such monitors were not available widely in clinical practice and they were difficult to use, especially in pediatric patients, rendering intraoperative quantitative neuromuscular monitoring challenging. The overall findings of our study provide important new information about pediatric patients’ hand size dimensions that will be helpful in the development of pediatric accessories such as monitor cables and skin surface electrodes used for quantitative (electromyographic) neuromuscular monitoring. The availability of pediatric neuromuscular monitoring accessories will encourage and facilitate the adoption of quantitative neuromuscular monitors in routine pediatric practice, resulting in safer perioperative anesthetic care. Although guidelines for quantitative neuromuscular monitoring in adults have been published in 2023 [3,4], these recommendations do not specifically address the pediatric anesthesia best practices.

It is now well recognized that postoperative residual neuromuscular block is an independent risk factor for the development of postoperative complications such as desaturation, pulmonary aspiration and pneumonia [7] in both pediatric and adult patients. Quantitative neuromuscular monitoring reduces the incidence of residual neuromuscular block regardless of reversal agent (neostigmine of sugammadex) [8]; reduces postoperative complications [9]; decreases the doses of agents needed for antagonism of neuromuscular block [10] and the cost of reversal agents [11,12]; and reduces hospital length of stay [5,9]. Additionally, appropriate intraoperative management of neuromuscular block improves surgical conditions to facilitate surgery [3].

To answer clinical needs, medical device manufacturers have started to develop new user-friendly quantitative monitors employing different technologies. In order to design appropriate sensors for children of different ages and facilitate their routine use, it is crucial to determine the dimensions of the hands, since quantitative monitoring relies on appropriate identification of the course of the ulnar nerve along the distal volar forearm, and the location of the target muscles (adductor pollicis, abductor digiti minimi, first dorsal interosseus muscles). However, pediatric anthropometric data to help design appropriate electrodes were scarce. To help the development of the pediatric strip electrode of an electromyography-based neuromuscular monitor (TetraGraph), we collected hand size dimensions and anthropometric data from children aged 1–15 years.

**Figure 2:**
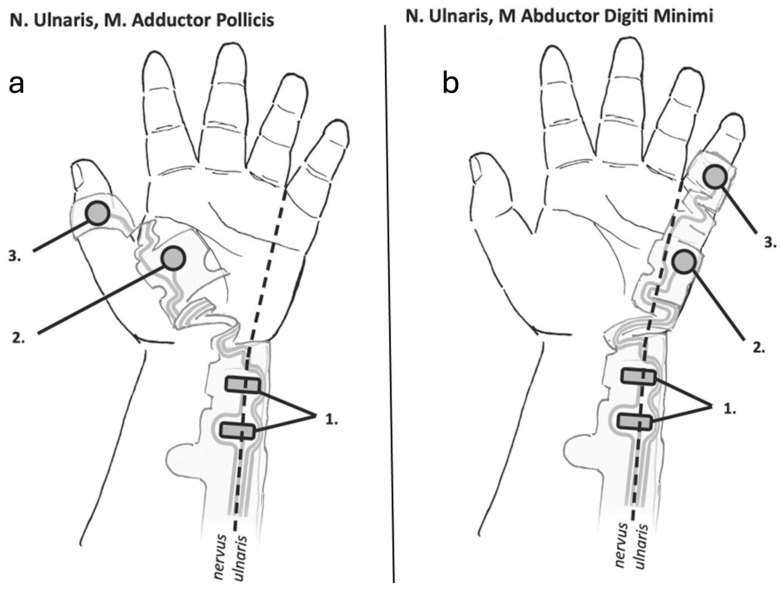
Typical electrode placement on the hand for electromyographic monitoring of the adductor pollicis (**a**) and abductor digiti minimi (**b**) muscles. (**a**) The stimulating electrodes (1) are placed along the ulnar nerve with the negative electrode distally. The sensing electrode (2) and reference electrode (3) are placed on the belly of the adductor pollicis muscle and the interphalangeal joint of the thumb, respectively. (**b**) The stimulating electrodes (1) are placed along the ulnar nerve with the negative electrode distally. The sensing electrode (2) and reference electrode (3) are placed on the belly of the abductor digiti minimi muscle and the interphalangeal joint of the 5th finger, respectively. Illustrations courtesy of Senzime AB (publ.), Uppsala, Sweden.

Electromyography measures the elicited cMAP amplitudes following stimulation of a peripheral nerve. The typical EMG electrode setting consists of one pair of stimulating and one pair of recording electrodes (Figure 2a,b). The positive and negative stimulating electrodes are placed along the course of the ulnar nerve, with the distal negative electrode placed 2–3 cm proximally to the wrist crease [1]; the positive electrode is placed on the skin 3–4 cm proximal to the negative electrode in adults, and 2 cm proximal to the negative electrode in children. The recording (active) electrode is placed on the belly of the target muscle (AP, ADM, or FDI), and the reference electrode is placed on the muscle’s insertion site, typically a distal joint.

The most common site for neuromuscular monitoring is the hand. The sensitivity of hand muscle to muscle relaxants is between the high resistance of the diaphragm (a central muscle) to the effects of neuromuscular blocking agents, and the high sensitivity of the pharyngeal muscles, which are the last to recover from neuromuscular block. Current guidelines for management of neuromuscular block [3,4] were based on studies in which responses of the adductor pollicis muscle to ulnar nerve stimulation were investigated.

Similar to the adductor pollicis muscle, the abductor digiti minimi muscle is also innervated by the ulnar nerve and has a similar recovery profile, although it may be slightly more resistant to muscle relaxants. Although the abductor digiti minimi may recover slightly faster from neuromuscular block, this difference is not clinically significant [13]. The abductor digiti minimi compound muscle action potential signals have been reported to be higher [14] and more consistent [13], while clinicians generally find placement of the electrodes along the medial side of the hand (along the abductor digiti minimi muscle) to be more intuitive. Additionally, the ADM electrode location is more resistant to movement and perspiration, reducing the likelihood of electrode displacement during longer surgical procedures. Therefore, the hypothenar (abductor digiti minimi) is a good alternative to the thenar (adductor pollicis) electrode placement for intraoperative monitoring.

The adult electrode array of the TetraGraph (TetraSens) was designed to monitor the function of the adductor pollicis, the first dorsal interosseous, or the abductor digiti minimi muscles. When designing pediatric strip electrodes, the manufacturer considered two options, either to design several sizes of electrodes for various age groups or to design a one-size-fits-all electrode. In order to develop sensors that would be most appropriate for newborns, infants and children, it was important to know how strong the correlation between hand size parameters and the age of the children is, or if there existed other anthropometric parameters that would be better indicators of this correlation. The alternative indicators were weight and height, as they are routinely recorded in the anesthesia records as standard of care and do not require extra entries from the anesthesia team.

Previous reports [15,16] described using EMG electrodes designed for adult patient use in a total of 100 pediatric patients aged 11 ± 3.0 years and weighing between 20–60 kg; their mean weight was 39.6 ± 10.8 kg. The authors of this report concluded, “although developed primarily for use in adults with an adult-sized sensor, our preliminary data suggest that the TetraGraph^TM^ EMG monitor can be used in pediatric patients as small as 20 kg.” However, the use of adult-sized electrodes in pediatric patients under 20 kg remained problematic, and development of smaller, pediatric electrodes was needed.

As expected, in our study, the hand size parameters increased according to the children’s age (Table 1). Height of the children showed closer correlation with hand size parameters (Pearson’s correlation coefficients: 0.702–0.961) than age (0.665–0.904) or weight (0.675–0.863, respectively). Correlation was weaker when the data were examined in the prespecified age groups, likely as a result of the relatively few datapoints in each group.

Our study, although novel, has certain limitations. Although complete datasets were obtained from 126 children, the number of children in each year group varied between 6 and 16. This likely limited the strength of the correlations between hand measurements and the children’s anthropometric data. Nevertheless, these measurements provide a good starting framework of pediatric sensor dimensions (total length, width, inter-electrode distance) for the development of future electromyographic sensors designed for intraoperative neuromuscular monitoring.

## 5. Conclusions

The presented hand dimension database has helped document the dimensions of the pediatric hand in children between the ages of 1 and 15 years, including forearm length and wrist circumference. This detailed information was not previously available and should help in the future design of electromyographic surface electrodes used in pediatric anesthesia care.

## Figures and Tables

**Table 1:** Specific parameters (dimensions) in the individual age groups.

Age (Years)	*n*	Weight (kg)	Height (cm)	Distance A (cm)	Distance B (cm)	Distance C (cm)	Distance D (cm)	Distance E (cm)	Forearm Length (cm)	Wrist Circumference (cm)
1–2	25	10.7 ± 1.6	79.8 ± 4.4	4.3 ± 0.4	3.2 ± 0.4	3.0 ± 0.3	4.1 ± 0.4	10.1 ± 0.6	11.9 ± 0.8	11.3 ± 1.0
2–3	32	12.8 ± 3.2	91.5 ± 7.6	4.5 ± 0.9	3.9 ± 0.8	3.3 ± 0.6	4.2 ± 0.7	11.0 ± 1.0	13.5 ± 1.7	11.6 ± 1.1
3–4	31	14.9 ± 2.8	98.8 ± 6.4	4.9 ± 0.6	4.0 ± 0.8	3.5 ± 0.4	4.7 ± 0.9	12.0 ± 0.8	14.9 ± 2.2	12.1 ± 1.1
4–5	22	17.2 ± 3.4	106.7 ± 5.4	5.3 ± 0.4	4.1 ± 0.3	3.5 ± 0.3	5.3 ± 0.7	12.5 ± 0.9	15.9 ± 1.3	12.1 ± 1.2
5–6	31	17.8 ± 2.9	111.1 ± 5.8	5.4 ± 0.4	4.3 ± 0.5	4.1 ± 0.9	5.0 ± 0.6	12.7 ± 0.9	16.9 ± 2.2	12.1 ± 0.8
6–7	12	26.5 ± 7.2	124.5 ± 5.7	6.1 ± 0.4	4.9 ± 0.4	4.3 ± 0.7	5.8 ± 0.5	14.8 ± 0.7	18.4 ± 0.8	13.6 ± 1.5
7–8	25	24.8 ± 3.5	125.2 ± 7.1	6.0 ± 0.6	4.7 ± 0.5	3.8 ± 0.4	6.0 ± 0.5	14.6 ± 0.8	19.2 ± 1.2	12.9 ± 0.7
8–9	30	29.5 ± 6.7	131.5 ± 8.1	6.4 ± 0.6	5.0 ± 0.7	4.3 ± 0.6	6.0 ± 0.8	14.9 ± 1.0	19.7 ± 1.1	13.6 ± 1.0
9–10	14	27.9 ± 6.9	132.9 ± 8.9	5.9 ± 0.5	5.0 ± 0.7	3.9 ± 0.6	6.1 ± 0.9	14.9 ± 1.3	20.7 ± 1.8	12.9 ± 2.7
10–11	16	39.3 ± 5.2	147.2 ± 7.2	6.6 ± 0.4	5.6 ± 0.5	4.1 ± 0.4	7.1 ± 0.6	16.9 ± 0.7	23.8 ± 1.7	14.5 ± 0.9
11–12	18	36.4 ± 12.7	141.8 ± 8.8	6.4 ± 0.9	5.3 ± 0.6	4.5 ± 0.6	6.6 ± 0.7	16.4 ± 1.4	22.0 ± 2.1	14.0 ± 1.4
12–13	18	48.7 ± 11.1	153.4 ± 8.3	6.4 ± 1.0	5.8 ± 0.6	4.7 ± 0.5	6.8 ± 1.1	16.9 ± 4.6	22.8 ± 4.5	15.0 ± 1.0
13–14	14	51.0 ± 12.1	162.9 ± 13.7	7.2 ± 0.4	6.1 ± 0.6	4.9 ± 0.4	7.4 ± 0.5	18.3 ± 1.4	24.9 ± 2.4	15.7 ± 1.0
14–15	12	63.8 ± 25.3	162.3 ± 13.5	7.2 ± 1.2	6.0 ± 0.7	4.9 ± 1.0	7.6 ± 0.5	18.1 ± 1.7	25.7 ± 3.0	16.5 ± 2.2

*n* = number of recorded datapoints.

**Table 2:** Pearson’s correlation coefficients of the demographic parameters and hand size parameters in the prespecified age groups.

		12–23 Months	2–5 Years	6–11 Years	12–15 Years	All Groups
Age (years)	Distance A	−0.12 (*n* = 24)	0.454 * (*n* = 114)	0.212 * (*n* = 115)	0.353 * (*n* = 44)	0.773 * (*n* = 297)
	Distance B	0.24 (*n* = 24)	0.202 * (*n* = 114)	0.339 * (*n* = 115)	0.174 (*n* = 44)	0.78 * (*n* = 297)
	Distance C	0.318 (*n* = 24)	0.426 * (*n* = 114)	0.188 * (*n* = 115)	0.15 (*n* = 44)	0.665 * (*n* = 297)
	Distance D	0.427 * (*n* = 24)	0.382 * (*n* = 114)	0.397 * (*n* = 115)	0.396 * (*n* = 44)	0.81 * (*n* = 297)
	Distance E	0.206 (*n* = 24)	0.561 * (*n* = 114)	0.532 * (*n* = 115)	0.329 * (*n* = 44)	0.904 * (*n* = 297)
Weight (kg)	Distance A	0.856 * (*n* = 24)	0.655 * (*n* = 114)	0.6 * (*n* = 115)	0.528 * (*n* = 44)	0.781 * (*n* = 297)
	Distance B	0.6 * (*n* = 24)	0.445 * (*n* = 114)	0.562 * (*n* = 115)	0.364 * (*n* = 44)	0.76 * (*n* = 297)
	Distance C	0.711 * (*n* = 24)	0.446 * (*n* = 114)	0.284 * (*n* = 115)	0.617 * (*n* = 44)	0.675 * (*n* = 297)
	Distance D	0.0262 (*n* = 24)	0.311 * (*n* = 114)	0.561 * (*n* = 115)	0.202 (*n* = 44)	0.736 * (*n* = 297)
	Distance E	0.575 * (*n* = 24)	0.634 * (*n* = 114)	0.812 * (*n* = 115)	0.496 * (*n* = 44)	0.863 * (*n* = 297)
Height (cm)	Distance A	0.771 * (*n* = 20)	0.423 * (*n* = 107)	0.632 * (*n* = 113)	0.518 * (*n* = 44)	0.821 * (*n* = 284)
	Distance B	0.624 * (*n* = 20)	0.339 * (*n* = 107)	0.679 * (*n* = 113)	0.753 * (*n* = 44)	0.845 * (*n* = 284)
	Distance C	0.616 * (*n* = 20)	0.526 * (*n* = 107)	0.327 * (*n* = 113)	0.505 * (*n* = 44)	0.702 * (*n* = 284)
	Distance D	−0.0706 (*n* = 20)	0.34 * (*n* = 107)	0.596 * (*n* = 113)	0.521 * (*n* = 44)	0.828 * (*n* = 284)
	Distance E	0.296 (*n* = 20)	0.725 * (*n* = 107)	0.871 * (*n* = 113)	0.907 * (*n* = 44)	0.961 * (*n* = 284)
Forearm length (cm)	Distance A	0.0296 (*n* = 24)	0.672 * (*n* = 114)	0.537 * (*n* = 115)	0.469 * (*n* = 44)	0.832 * (*n* = 297)
	Distance B	−0.276 (*n* = 24)	0.451 * (*n* = 114)	0.638 * (*n* = 115)	0.536 * (*n* = 44)	0.825 * (*n* = 297)
	Distance C	0.122 (*n* = 24)	0.291 * (*n* = 114)	0.264 * (*n* = 115)	0.551 * (*n* = 44)	0.668 * (*n* = 297)
	Distance D	0.5 * (*n* = 24)	0.431 * (*n* = 114)	0.666 * (*n* = 115)	0.657 * (*n* = 44)	0.843 * (*n* = 297)
	Distance E	0.553 * (*n* = 24)	0.661 * (*n* = 114)	0.12 (*n* = 115)	0.846 * (*n* = 44)	0.312 * (*n* = 297)
Wrist circumference (cm)	Distance A	0.594 * (*n* = 24)	0.377 * (*n* = 114)	0.483 * (*n* = 115)	0.576 * (*n* = 43)	0.733 * (*n* = 296)
	Distance B	0.481 * (*n* = 24)	0.395 * (*n* = 114)	0.344 * (*n* = 115)	0.427 * (*n* = 43)	0.707 * (*n* = 296)
	Distance C	0.565 * (*n* = 24)	0.408 * (*n* = 114)	0.099 (*n* = 115)	0.542 * (*n* = 43)	0.608 * (*n* = 296)
	Distance D	−0.208 (*n* = 24)	0.149 (*n* = 114)	0.376 * (*n* = 115)	0.262 (*n* = 43)	0.658 * (*n* = 296)
	Distance E	0.223 (*n* = 24)	0.483 * (*n* = 114)	0.106 (*n* = 115)	0.511 * (*n* = 43)	0.267 * (*n* = 296)

*n* = number of recorded datapoints. * *p* < 0.05.

**Table 3:** Analysis of the differences between the 4 grouped age bins (1–2 years old; 2–5 years old; 6–11 years old; and 12–15 years old).

Variable	F-Stat	*p*-Value	eta-Squared
Weight (kg)	235.81	*p* < 0.001	0.7050
Height (cm)	477.89	*p* < 0.001	0.8289
Distance A (cm)	142.68	*p* < 0.001	0.5912
Distance B (cm)	155.17	*p* < 0.001	0.6113
Distance C (cm)	61.13	*p* < 0.001	0.3826
Distance D (cm)	171.10	*p* < 0.001	0.6343
Distance E (cm)	234.40	*p* < 0.001	0.7038
Forearm length (cm)	284.37	*p* < 0.001	0.7424
Wrist circumference (cm)	113.51	*p* < 0.001	0.5350

kg = kilogram. cm = centimeters.

## Data Availability

The original contributions presented in this study are included in the article. Further inquiries can be directed to the corresponding author.
